# Quorum Quenching-Guided Inhibition of Mixed Bacterial Biofilms and Virulence Properties by Protein Derived From Leaves of *Carissa carandas*

**DOI:** 10.3389/fcimb.2022.836819

**Published:** 2022-07-14

**Authors:** Manjari Shukla, Vineeta Singh, Hamza Habeeballah, Mustfa F. Alkhanani, Manjul Lata, Yusuf Hussain, Madhuparna Mukherjee, Mukesh Pasupuleti, Abha Meena, Bhartendu Nath Mishra, Shafiul Haque

**Affiliations:** ^1^ Department of Biotechnology, Institute of Engineering and Technology, Dr. A.P.J. Abdul Kalam Technical University, Lucknow, India; ^2^ Faculty of Applied Medical Sciences in Rabigh, King Abdulaziz University, Rabigh (Jeddah), Saudi Arabia; ^3^ Emergency Medical Services Department, College of Applied Sciences, AlMaarefa University, Riyadh, Saudi Arabia; ^4^ Microbiology Division, CSIR, Central Drug Research Institute, Lucknow, India; ^5^ Microbiology Division, Academy of Scientific and Innovative Research (AcSIR), Ghaziabad, India; ^6^ Bioprospection and Product Development Division, CSIR, Central Institute of Medicinal and Aromatic Plants, Lucknow, India; ^7^ Centre for Nanosciences, Indian Institute of Technology, Kanpur, India; ^8^ Research and Scientific Studies Unit, College of Nursing and Allied Health Sciences, Jazan University, Jazan, Saudi Arabia

**Keywords:** biofilm, nosocomial infection, quorum sensing, quorum quenching, virulence

## Abstract

The inhibition/degradation potential of *Carissa carandas* proteinaceous leaf extract against mixed bacterial biofilm of *Staphylococcus aureus* MTCC 96, *Escherichia coli* MTCC 1304, *Pseudomonas aeruginosa* MTCC 741, and *Klebsiella pneumoniae* MTCC 109, responsible for nosocomial infections, was evaluated. Distinct inhibition/degradation of mixed bacterial biofilm by the proteinaceous leaf extract of *C. carandas* was observed under a microscope, and it was found to be 80%. For mono-species biofilm, the maximum degradation of 70% was observed against *S. aureus* biofilm. The efficiency of aqueous plant extracts to inhibit the mono-species biofilm was observed in terms of minimum inhibitory concentration (MIC), and the best was found against *P. aeruginosa* (12.5 μg/ml). The presence of flavonoids, phenols, and tannins in the phytochemical analysis of the plant extract suggests the main reason for the antibiofilm property of *C. carandas*. From the aqueous extract, protein fraction was precipitated using 70% ammonium sulfate and dialyzed. This fraction was purified by ion-exchange chromatography and found to be stable and active at 10°C (pH 7). The purified fraction showed less than 40% cytotoxicity, which suggests that it can be explored for therapeutic purposes after in-depth testing. In order to investigate the mechanistic action of the biofilm inhibition, the plant protein was tested against *Chromobacterium violaceum* CV026, and its inhibitory effect confirmed its quorum quenching nature. Based on these experimental analyses, it can be speculated that the isolated plant protein might influence the signaling molecule that leads to the inhibition effect of the mixed bacterial biofilm. Further experimental studies are warranted to validate our current findings.

## Introduction

Biofilm is an aggregation of microbes formed on biotic and abiotic surfaces, which is a worldwide problem due to its resistant nature towards biocides, antimicrobials, antibiotics, etc. This nature is due to its architecture and mechanism. Several antimicrobial molecules discovered earlier fail to penetrate the biofilms because of the extracellular polymeric substances (EPS) that act as a barrier and protect the microbial cells (Singh et al., 2015; Phuengmaung et al., 2021). Biofilms cause various infections to humans, and they are harmful to industries and household systems. In the past decade, there has been an amplified focus on the collection of clinically significant bacteria known for their potential of biofilm formation, *viz*., *Staphylococcus aureus*, *Enterococcus faecalis*, *Pseudomonas aeruginosa*, *Acinetobacter baumannii*, *Klebsiella pneumoniae*, and *Enterobacter* species, also termed as ESKAPE pathogens, as they are capable of escaping from the *cidal* action of many antibiotics (Gupta and Birdi, 2017).

Quorum-sensing molecules, also known as autoinducers, play an important role in biofilm formation. They are small, diffusible, extracellular signal molecules that are diverse in nature. Gram-negative and Gram-positive bacteria have *N*-acyl-homoserine lactone (AHL) and oligopeptides signaling molecules, respectively. In interspecies communication, autoinducer-2 works as an autoinducer (Haque et al., 2019; Wu et al., 2004). Quorum sensing regulates various activities in bacteria like antibiotic resistance and virulence factors. There are two main components in the quorum-sensing mechanism: signal molecules and receptors. Signal molecules or autoinducers (bacterial quoromone) are small, diffusible, extracellular signal molecules that range from low to high molecular weight. The first signal molecule was identified in the marine bacterium *Vibrio fischeri*, which induces luminescence properties in that organism (De la Fuente-Núñez et al., 2013). Quorum sensing is involved in biological functions like luminescence, pigment production, conjugation, motility, antibiotic production, virulence, and biofilm formation (Dong and Zhang, 2005; Dickschat, 2010).

All processes that are involved in the interference of the quorum-sensing phenomenon are named quorum quenching (QQ), also called signal interference or antipathogenic approach. QQ is an alternative approach to overcome the problem of drug-resistant microbes because it does not affect the growth of microbes directly. QQ molecules are diverse in nature, mode of action, and target. QQ molecules are of variety in nature (chemical compounds and enzymes), mode of action (quorum-sensing signal degradation, inhibition, competitive inhibition, and so on), and targets because all steps in the quorum-sensing mechanism of microbes like synthesis, diffusion, accumulation, and perception of signals may be affected. Physical parameters like pH and temperature can also affect the half-life of quorum-sensing signals. Chemicals that interfere with the quorum-sensing pathway and enzyme, which inactivate quorum-sensing signals, are called quorum-sensing inhibitors and QQ enzymes, respectively (Flemming and Wingender, 2010).

While keeping the significant structural role of EPS and quorum-sensing phenomenon for the development of biofilm, and efficiency of plant-based molecules to combat this condition in view, the present study was started with the quest to find out the plants that have been reported for their anti-biofilm activity, and literature survey supported that many Indian medicinal plants possess the same. Overall, this study focuses on the purification and characterization of plant-derived quorum quenchers. Thus, the objectives of this study were to analyze various mono-species and mixed biofilms, isolation of plant metabolites showing QQ behavior, and biofilm inhibition and degradation studies by plant protein(s).

## Materials and Methods

### Microbial Strains and Chemicals Used

Five bacterial strains, *Escherichia coli* MTCC 1304, *P. aeruginosa* MTCC 741, *K. pneumoniae* MTCC 109, *S. aureus* MTCC 96, and *Chromobacterium violaceum* CV026, were used in the study. All bacterial strains except *C. violaceum* were maintained in nutrient agar (NA) slant at 4°C, and *C. violaceum* was maintained in trypticasein soy (TS) broth slant at 4°C. The culture was inactivated prior to any test at 37°C and 27°C.

All the chemicals used for different experiments and resazurin dye (7-hydroxy-10-oxidophenoxazin-10-ium-3-one, sodium) were purchased from HiMedia Chemicals Ltd, Mumbai, India.

### Biofilm Formation

Biofilm of Gram-negative (*E. coli* MTCC 1304, *P. aeruginosa* MTCC 741, and *K. pneumoniae* MTCC 109), Gram-positive (*S. aureus* MTCC 96), and the mixed strain was formed on petri plates filled with glass wool. One percent of the overnight-grown culture of each bacterium was used as an inoculum, added to 10 ml of fresh media, inoculated on 5-g glass wool, and kept undisturbed for 2 days at 37°C. The biofilm grown on glass wool was visually observed after 48 h. For mixed biofilm, 25% volume of each fresh bacterial culture was added on glass wool and kept for 5 days at 37°C under static conditions. The mixed biofilm formed on glass wool was visually observed, and EPS extraction from the cultured media was performed.

### Biofilm Inhibition and Degradation Analysis

Biofilm inhibition and degradation tests were performed in 96-well microtiter plates as described by Kalia et al. (2015), with minor modifications, and crystal violet (CV) dye was used for the quantification of the biofilm. In a 96-well plate, 100 µl of the freshly prepared bacterial suspension (0.5 McFarland’s standard) and 100 µl of 2 mg/ml stock of the plant extract was added and incubated for 48 h at 37°C. After the incubation medium was removed, wells were washed with distilled water to remove planktonic cells. Further 200 µl of 0.4% CV dye was added and incubated for 20 min to stain the attached biofilm followed by washing the wells with distilled water to remove the extra dye, and then plates were dried. The biofilm was re-solubilized with dimethyl sulfoxide (DMSO) (99.5%) and then read with a spectrophotometer at 620 nm in an ELISA reader. The percentage of biofilm inhibited was calculated by the following formula.


$$\%\;of\;Biofilm\;inhibition=\frac{{\text{OD}}_{620\text{nm}}\text{ in control}-{\text{OD}}_{620\text{nm}}\text{ in treatment}}{{\text{OD}}_{620\text{nm}}\text{ in control}}\times 100$$

Biofilm degradation assay was performed as explained by Perumal and Mahmud (2013), with minor modifications. The 24-h-old biofilm was formed in 96-well plates, and after incubation, the medium was removed. Adherent biofilm was treated with 100 µl of plant extract (2 mg/ml of stock solution) and incubated for 24 h at 37°C. The CV dye was added after washing the wells with distilled water and was kept for 20 min for staining purposes. After staining, the extra dye was removed by washing, and the plate was dried. Further DMSO was added to resolubilize the biofilm, and plates were read at 620 nm in an ELISA plate reader. All the experiments were performed in triplicate. The degradation percentage was calculated by the following formula:


$$\%\;of\;Biofilm\;degradation=\frac{{\text{OD}}_{620\text{nm}}\text{ in control}-{\text{OD}}_{620\text{nm}}\text{ in treatment}}{{\text{OD}}_{620\text{nm}}\text{ in control}}\times 100$$

### Extracellular Polymeric Substance Extraction and Its Analysis

For EPS production in mixed and individual bacterial biofilm, an inoculum of bacterial strains, *S. aureus*, *P. aeruginosa*, *E. coli*, and *K. pneumoniae*, was prepared in 15 ml of nutrient broth in petri-plates with 0.5-g glass wool in it. The treatment of biofilm was done with plant protein after 48 h. The plates were incubated at 37°C for 5 days under static conditions. The steps involved in the extraction of EPS from 5-day-old culture medium were performed as described by Gong et al. (2009) and are depicted as follows, Various microbes’ inoculated culture was incubated for 5 days at 37°C and then heat-inactivated at 100°C for 10 min. The culture was centrifuged at 10,000 rpm for 10 min, and the supernatant was precipitated with cold ethanol (double volume). After overnight incubation at 4°C, the mixture was centrifuged at 10,000 rpm for 25 min in a cooling centrifuge, and the obtained pellet was dissolved in distilled water. Total carbohydrate, reducing sugars, and the total soluble protein were estimated by various analytical methods.

### Natural Product Extraction and Purification to Study Biofilm Inhibition/Degradation

Various plants (**Table SI1**) were collected freshly from the Institute of Engineering and Technology Campus, Sitapur Road, Lucknow (26.9143°N, 80.9410°E), and the local market of Alambagh, Lucknow (Uttar Pradesh), India (26.8128°N, 80.9014°E). The plant parts were collected fresh, washed, shade dried, and powdered. Each plant sample measuring 2 g was dissolved in 20 ml of methanol and 20 ml of aqueous solvent (1:10 w/v distilled water) and kept at a rotary shaker overnight. Flask was kept in a stable position for 1 h, and then extract was filtered through Whatman No. 2 filter paper, was concentrated to dry crude, and stored in a sterile Eppendorf tube at 4°C. The methanolic and aqueous plant extracts were used to test the existence of various phytochemicals like flavonoids, glycoside, phenol, amino acids, saponins, tannins, quinone, coumarins, carbohydrate, alkaloids, and terpenoids using the standard protocols (Goyal et al., 2016; Sánchez et al., 2016; Santhi and Sengottuvel, 2016) (**Table SI2**).

### Purification of Plant Protein

The protein content present in the aqueous medium of the plant extract was isolated according to the steps mentioned by Gangwar et al. (2016), with minor modifications.

#### Protein Precipitation by Ammonium Sulfate Precipitation Method

The protein contents of the aqueous extract of the plant samples were precipitated with ammonium sulfate while stirring at a magnetic stirrer to get 70% saturation at 4°C. The flask was kept overnight at a magnetic stirrer. The samples were centrifuged for 15 min at 10,000 rpm, and the protein precipitate as the pellet was stored in 0.1 M of phosphate buffer (7 pH) at 4°C.

#### Dialysis of the Precipitate to Remove Impurities

The sample (precipitate in buffer) was taken in the dialysis membrane (12 kDa of cutoff membrane). It was dialyzed in 0.1 M of phosphate buffer (pH 7) overnight on the stirrer at 4°C. The buffer was changed as per the requirement. Following the dialysis, QQ activity and phytochemical test of the plant aqueous extract, filtrate, precipitate, and dialyzed sample were determined.

#### Ion-Exchange Chromatography

The dialyzed sample was loaded on a pre-equilibrated (0.1 M of phosphate buffer) diethyl aminoethyl (DEAE) cellulose column. The unbound protein was washed with phosphate buffer, and the enzyme was eluted with an increasing concentration of NaCl (0.1–1.0 M) in 0.1 M of phosphate buffer at a flow rate of 0.5 ml/min. Fractions of 5 ml were collected and tested for protein content by Lowry assay.

### Protein Characterization

Liquid chromatography–tandem mass spectrometry (LC-MS) analysis of the sample was done with a C-18 250 × 4.6 mm column (Waters Instrument, Milford, MA, USA).

To analyze the sample, sodium dodecyl sulfate–polyacrylamide gel electrophoresis (SDS-PAGE) was performed using Mini-PROTEAN apparatus (Bio-Rad Laboratories Inc., Hercules, CA, USA) following the protocol of Laemmli et al. (1970). The gel was made with urea and other solutions as done with SDS–PAGE. The sample solution was prepared with Bio-Rad AG501-X6 mixed bed resin. Methyl green was used as a tracking dye, and peptide markers were used. The gel was run at 110 V for 2–3 h. The gel was stained with a staining solution containing Coomassie Brilliant Blue (CBB) 250 dye overnight and was destained with a destaining solution.

### Activity Analysis of Plant Protein

#### Determination of Minimum Inhibitory Concentration

Minimum inhibitory concentration (MIC) is the minimum concentration of the extract, which checks the visible growth of microbes. The assay was performed by the micro broth dilution method explained by Novy et al. (2015), with minor modifications, and the addition of resazurin dye. In a 96-well microtiter plate, 100 µl of bacteria inoculated in Luria–Bertani (LB) was added. Further 100 µl of plant extract was added to each first well of the row, and further dilution was done. Plates were incubated at 37°C for 24 h. After incubation, 20 µl of 0.01% resazurin dye was added and incubated for 30 min. Plates were read at 630 nm in an ELISA plate reader, and MIC was determined. The development of pink color indicated the growth of microbes, while blue color indicated inhibition of the bacterial growth.

#### Cytotoxicity Testing of Plant Extract

All the chemicals used in cell culture and MTT (methylthiazolyldiphenyl-tetrazolium bromide) assay, including MTT dye and doxorubicin, were purchased from Sigma-Aldrich (St. Louis, MO, USA). Human embryonic kidney epithelial adherent cell line, i.e., HEK293T, was purchased from ATCC (India). The MTT assay on protein samples of the plant was performed according to the protocol reported by Singh et al. (2020) and Khamwut et al. (2019). Before the MTT assay, 22,000–25,000 HEK293T cells were seeded in a 96-well plate, and treatment of different doses of plant extract was given for 24 h. Different stock solutions like 20, 5, and 1 mg/ml of the test drug (plant chemicals) were prepared so that the concentration of DMSO was kept below 0.5%. After the treatment, 10 µl of MTT dye (5 mg/ml) was added to each well already containing 100 µl of media, and plates were incubated in the dark for 4 h at 37°C. Afterward, the dye was discarded, 100 µl of DMSO was added to each well, and absorbance was recorded at 570 nm using a spectrophotometer (Multiskan™ Go SkanIt Software 4.0 version; Thermo Fisher Scientific, Waltham, MA, USA). Doxorubicin was taken as standard.

#### Effect of Different Factors on Plant Protein Activity

The dialyzed sample was characterized with respect to its optimal temperature, pH, solvent, and metal ion and surfactants for its activity. The optimum temperature for its anti-quorum-sensing activity was determined by checking the anti-quorum-sensing ability of the compound at different temperatures ranging from 4°C to 60°C at pH 7 in phosphate buffer (0.1 M) by using *C. violaceum* strain. Similarly, the optimum pH was determined using dialyzed sample dissolved in different pH values of phosphate buffer (4–10) of 0.1 M concentration and incubated for 10–12 h, and then anti-quorum activity was checked by agar well diffusion method. The effect of metal ions like Ca^2+^ (CaCl_2_), Mg^2+^ (MgCl_2_), Cu^2+^ (CuSO4), Fe^3+^ (FeCl_3_), SDS, and EDTA of concentration 0.01 M, incubated for 1 h, was observed by calculating and comparing its anti-quorum-sensing zone of inhibition. The stability of the protein sample in different solvents (ethanol, methanol, and DMSO) was observed by incubating the protein sample in solvents for 1 h and then comparing its anti-quorum activity with the control.

### Microscopic Analysis of the Effect of the Selected Plant Extract on Biofilms

The 24-h-old bacterial cultures were inoculated in fresh nutrient broth media. Bacterial culture measuring 5 ml was dispensed in petri dishes and then incubated at 37°C for 24 h. Biofilms were prepared on sterilized coverslips kept in petri dishes. After incubation, planktonic cells were discarded by pipetting out the media, and coverslips were rinsed with 1× phosphate-buffered saline (PBS) twice. Coverslips were stained with 0.4% CV dye for 15 min. Coverslips were rinsed twice for extra dye removal. Then coverslip was kept on a glass slide and observed under a light microscope at 100× for visualization of biofilms.

### Evaluation of Anti-Quorum-Sensing Activity of Plant Extracts as a Mechanistic Approach for Biofilm Inhibition/Degradation

The anti-quorum-sensing activity (QQ) of plant extracts was evaluated by using biosensor bioassay with *C. violaceum* strain by using the agar well diffusion method. A lawn of bacteria was formed on an agar plate by using freshly prepared culture in an LB medium. The wells were inoculated with 100 µl of stock solution of 2 mg/ml of aqueous and methanolic plant extract. Plates were incubated at 27°C for 48 h. Methanol (99%) and distilled water were used as a negative control. The anti-quorum-sensing activity was determined by the zone of inhibition (measured in mm) formed around the wells.

### Statistical Analysis

All the experiments were performed in triplicate, and the mean value of the data was presented. The statistical significance of the data was analyzed through one-way ANOVA by setting the significance level of p-value <0.05.

## Results

### Purification of Plant Protein

**Figure 1:**
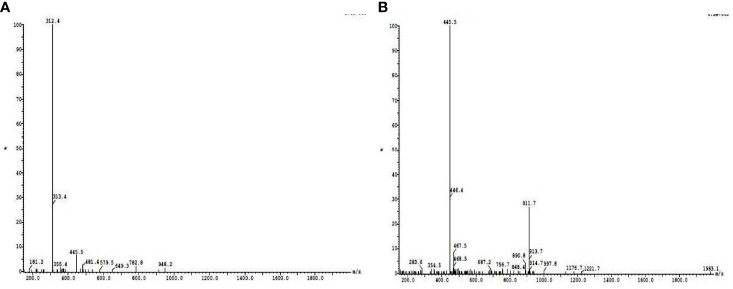
Liquid chromatography–tandem mass spectrometry (LC-MS) analysis of active column passed fractions collected at retention times of 19.88 **(A)** and 20.96 **(B)**.

The dialyzed sample was subjected to DEAE cellulose ion-exchange chromatography, and the fractions collected from the column were examined for the presence of protein by Lowry assay. The maximum protein content was eluted with 0.1 M of NaCl. All the fractions were subjected to anti-quorum-sensing activity, and the fractions showing positive activity were subjected to phytochemical activity. The results of phytochemical activities suggest that except quinone and coumarins, all the tested phytochemicals were present in the aqueous extract of the plant leaves. However, with the progress in the purification steps, their number reduces, and finally, in the dialyzed sample, only phenol, terpenoid, protein, saponin, and reducing sugar were present (**Table 1**). LC-MS analysis revealed that at retention times of 19.88 and 20.96, basic peaks were observed, further molecular mass of compounds was determined, and 312.5 *m*/*z* and 445 *m*/*z* were found to be present in maximum concentration (**Figure 1**). Further analysis suggested the presence of amino acids like aspartic acid, phenylalanine, and glutamic acid.

**Table 1:** Phytochemical testing of different extracts of plant *Carissa carandas*.

Phytochemical Extract	Car	Flv	Gly	Phe	Cou	Tan	Ter	Qui	Prt	Ps	Sap	RS
Filtrate	+	+	+	+	−	−	+	−	+	−	+	+
Precipitate	−	−	+	+	−	−	+	−	+	−	+	+
Dialyzed sample	−	−	−	+	−	−	+	−	+	−	+	+
Column Fractions	−	−	−	+	−	−	−	−	+	−	−	+

Car, carbohydrate; Flv, flavonoid; Gly, glycoside; Phe, phenol; Cou, coumarin; Tan, tannin; Ter, terpenoid; Qui, quinone; Prt, protein; Ps, phytosterol; Sap, saponin; RS, reducing sugar.

### Effect of Different Factors on Plant Protein Activity

**Figure 2:**
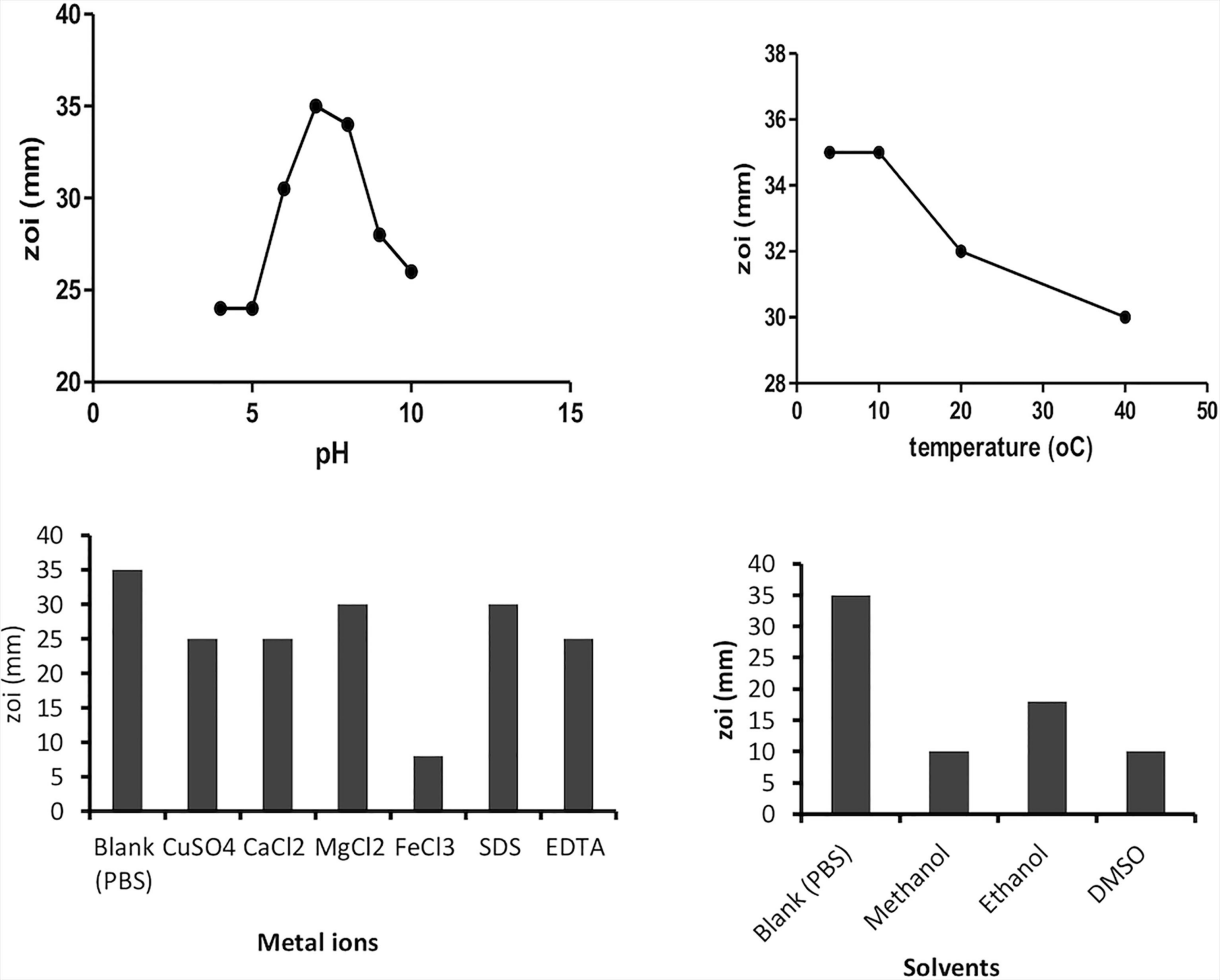
Effect of various factors (temperature, pH, solvents, and metal ions) on plant protein activity.

The plant protein extract was tested for having maximum anti-quorum-sensing activity at different physiochemical conditions like temperature, pH, solvents, metal ions, and surfactants. Under examined incubation temperatures (4°C–60°C), the protein retains the maximum anti-quorum-sensing activity between 4°C and 10°C. However, 80% of its activity is retained up to 40°C, which is in accordance with some previous reports that suggest plant proteins are stable up to 50°C temperature. Similarly, pH 7 (phosphate buffer) was found most suitable for protein stability. Metal ions like Mg^2+^ and surfactant EDTA were showing a positive effect on the isolated protein fraction. Plant sample dissolved in aqueous solvent was more active than in other solvents like methanol, ethanol, and DMSO; this suggests that the solvents lead to denaturation of the isolated protein resulting in the decrease of activity (**Figure 2**).

### Activity Analysis of Plant Protein

#### Minimum Inhibitory Concentration Determination

MIC determination of the selected plant extract was done by using resazurin dye, which changes color from pink to blue. A pink color indicates microbial growth, while blue color indicates inhibition of growth. Antibiotics chloramphenicol, ampicillin, and cefotaxime were taken as a positive control. The MIC value was found minimum (12.5 µg/ml) against *P. aeruginosa* (**Table 2**). MIC of plant protein was shown in **Table 2**. The MIC value of *Carissa carandas* aqueous leaf extracts was 100 μl against *S. aureus*, *K. pneumoniae*, and *C. violaceum* while 50 μl against *E. coli* and mixed microbes.

**Table 2:** Minimum inhibitory concentration of the selected plant protein (in μg/ml).

Microorganism	Chloramphenicol (D1)	Ampicillin (D2)	Cefotaxime (D3)	*Carissa carandas*
*Staphylococcus aureus*	25	100	12.5	100
*Klebsiella pneumoniae*	12.5	25	12.5	100
*Escherichia coli*	6.25	6.25	100	50
*Pseudomonas aeruginosa*	6.25	6.25	50	12.5
*Chromobacterium violaceum*	6.25	6.25	25	100
Mixed biofilm	12.5	12.5	100	50

#### Cytotoxicity Testing of Selected Plant Extract

**Figure 3:**
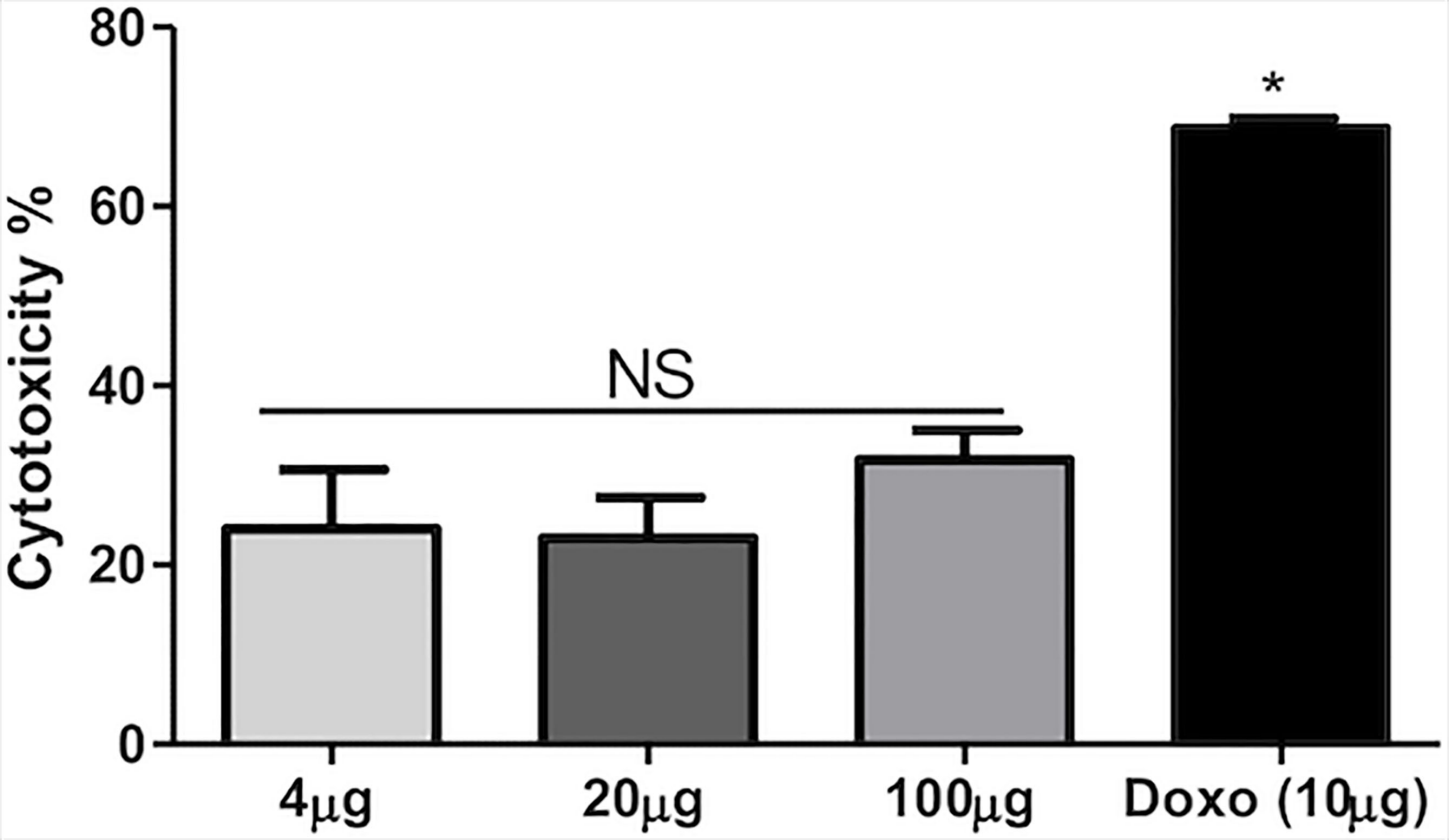
Percentage cytotoxicity vs. concentration of extract. NS = Non significant; * = Significant.

For the assessment of the cytotoxicity of plant extract, MTT assay was performed on HEK293T cells at three different doses at 100, 20, and 4 μg/ml along with positive control doxorubicin (10 μg/ml). Concentration of 100 μg/ml produced cytotoxicity above 30%, whereas the remaining concentrations at 4 and 20 μg/ml produced lower than 30% cytotoxicity. Statistically, there is a significant difference between the different concentrations of plant extract and standard drugs. Thus, the MTT assay suggests the non-toxic behavior of the plant extract against HEK293T cells and suggests that it can be used as a drug after detailed analysis. The percent cytotoxicity versus concentration of extract is shown in **Figure 3**. **Figure 3** depicts MTT data, wherein it was observed that at all the tested concentrations, i.e., 100, 20, and 4 μg/ml, and % cytotoxicity levels were almost similar (non-significant). Hence, the tested peptides are non-toxic even at 100 µg/ml based on the data.

### Biofilm Inhibition and Degradation Analysis

The plant extract of *C. carandas* was showing significant inhibition and degradation percentage, and the results are presented in **Table 3**. The plant extract was found to show 80% inhibition against Gram-positive strains while 60% inhibition against Gram-negative strains. The degradation percentage of biofilm by plant extract was found at 70% and 60% against Gram-positive and Gram-negative strains, respectively. Ampicillin (D2) and cefotaxime (D3), each measuring 2 mg/ml, were also taken as standard.

**Table 3:** Percentage inhibition (I) and degradation (D) observed by the plant extract of *Carissa carandas*.

Microorganism	*Klebsiella pneumoniae*		*Pseudomonas aeruginosa*		*Staphylococcus aureus*		*Escherichia coli*		*Chromobacterium violaceum*		Mixed	
	I	D	I	D	I	D	I	D	I	D	I	D
Ampicillin (D2) (100 μl)	94	44	90	34	88	64	90	36	95	78	90	67
Ampicillin (D2) (50 μl)	80	22	80	22	65	76	77	78	90	65	82	40
Cefotaxime (D3) (100 μl)	96	43	92	48	90	57	90	72	92	73	92	82
Cefotaxime (D3) (50 μl)	80	33	80	48	77	40	85	69	87	65	78	60
Compound (100 μl)	50	40	63	28	80	70	78	66	90	60	80	60
Compound (50 μl)	42	32	50	10	70	60	66	50	80	48	72	50

Biofilm inhibition and degradation analysis of the selected plant extract (C) were done by using a 96-well microtiter plate and CV dye. The inhibition percentage of 48-h-old biofilm in the presence of selected plant extract was compared with the control adherent biofilm without treatment. Antibiotics like ampicillin (D2) and cefotaxime (D3), each measuring 2 mg/ml, were also taken as standard. The percentage inhibition and degradation are tabulated in **Table 3**. All the values were examined by one-way ANOVA using GraphPad Prism 7 software, and p-value >0.5 was considered significant.

Through the present study, it can be concluded that the selected plant compound was capable of inhibiting biofilm formed by Gram-positive microbes up to 80% while by Gram-negative microbes up to 60% at 100 μl of concentration. The selected plant extract has the potential to degrade biofilms formed by Gram-positive microbe up to 70% and by Gram-negative microbes up to 60% at 100 μl of concentration.

### Microscopic Analysis of Biofilm

**Figure 4:**
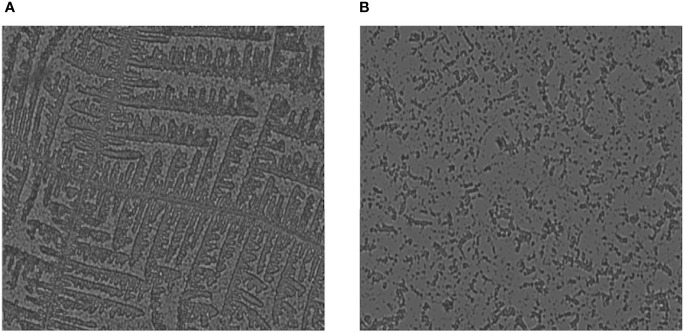
Fluorescence microscopic image showing degradation of biofilm when treated with the protein extract of *Carissa carandas* leaves. **(A)** Control (non-treated). **(B)** After 24-h treatment.

Biofilm on coverslips was made with *E. coli*, *P. aeruginosa*, *S. aureus*, *K. pneumoniae*, and *C. violaceum* bacterial strains and was visualized under bright-field microscopy. By examining the microbial colonies under a microscope, it was observed that the microbial colonies that were treated after 24 h had deformed and had fewer colonies as compared to non-treated ones (**Figure 4**). This endorses that the plant extract has microbial colony-degrading capability. The biofilm has a network-like appearance when seen under the microscope, while in the bacterial samples where the plant extract was applied, the degraded network was observed with fewer colonies in number.

### Extracellular Polymeric Substance Extraction and Analysis

**Figure 5:**
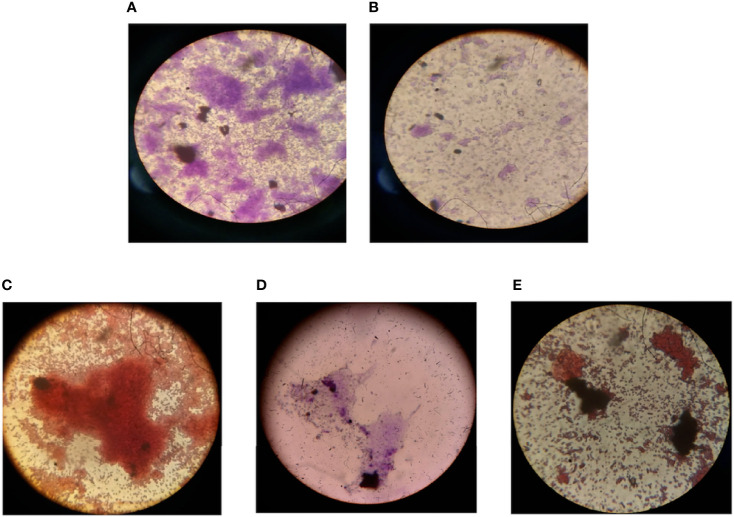
Microscopic analysis of the effect of compounds on mixed biofilm. **(A)** Biofilm formed by mixed microbes (S.a, K.p, E.c, P.a, and C.v). **(B)** Biofilm formation inhibition by selected compound extracellular polymeric substances (EPS) extraction and analysis. **(C)** Carbohydrate. **(D)** Protein. **(E)** Lipid content of the biofilm EPS. S.a, *Staphylococcus aureus*; K.p, *Klebsiella pneumoniae*; E.c, *Escherichia coli*; P.a, *Pseudomonas aeruginosa*; C.v, *Chromobacterium violaceum*.

EPS of the biofilm was extracted by ethanol precipitation method and analyzed according to the carbohydrates, proteins, and lipid staining using iodine, ninhydrin, and Sudan black B dye, respectively. The slides were visualized under 100× oil immersion in a light microscope, the presence of the mentioned components was observed (**Figure 5**). Further, in the quantitative analysis, the control biomass (10.85 g) and EPS (765 mg) were found to decrease nearly 40% after the treatment with the protein extract of the *C. carandas* leaves (**Table 4**).

**Table 4:** Effect of selected plant extract on the EPS inhibition and analysis of its content.

Microorganisms	Without treatment					With treatment					
	*K.p*	*P.a*	*E.c*	*S.a*	Mixed	*K.p*	*P.a*	*E.c*	*S.a*	Mixed
Biomass (g)	12.38	12.80	14.00	14.38	12.978	11.28	11.71	12.74	8.688	10.048
EPS (mg)	60	148	310	200	410	40	100	200	150	300
Total carbohydrate (mg/ml)	0.142	0.222	0.217	0.227	0.223	0.097	0.168	0.170	0.187	0.180
Total reducing sugar (mg/ml)	0.030	0.019	0.014	0.090	0.081	0.020	0.010	0.018	0.040	0.015
Total protein (mg/ml)	0.373	0.40	0.220	0.19	0.304	0.23	0.35	0.20	0.10	0.23

K.p, Klebsiella pneumoniae; P.a, Pseudomonas aeruginosa; E.c, Escherichia coli; S.a, Staphylococcus aureus; C.v, Chromobacterium violaceum; EPS, extracellular polymeric substances.

### Role of Anti-Quorum-Sensing Activity of Plant Extracts in Biofilm Degradation

Methanolic and aqueous extracts of *C. carandas* leaves were tested for their anti-quorum-sensing activity against *C. violaceum*. The methanolic and aqueous extracts showed anti-quorum sensing with the zone of inhibition of 10 and 20 mm, respectively. The aqueous extract showed better activity than methanolic extract and suggests the proteinaceous nature of the compound, which was further confirmed by the protein assay. The results were further validated by biochemical activity at each step of protein purification.

## Discussion

The hazardous biofilms affect agriculture, food, environment, and medicines which can have various adverse effects on human health (Perumal and Mahmud, 2013; Ahn et al., 2018). Therefore, with the aim of exploration of the potential antimicrobial as well as antibiofilm agents, the present study was performed. From the screening process, leaf extract of *C. carandas* showing promising antimicrobial activity was selected for the studies performed to observe the mixed biofilm inhibition/degradation and QQ properties. In literature, several reports mentioned the inhibitory activity of plant extract towards pathogens. Khan et al. (2009) reported a 17-mm zone of inhibition with clove oil against *C. violaceum* CV026 strain. Similarly, Al-Haidari et al. (2016) reported that DMSO extract of *Allium sativum* possesses an anti-quorum-sensing activity. Recently, Tang et al. (2020) have reported *Hizikia fusiforme* for its antimicrobial and anti-quorum-sensing activities due to phlorotannins present in this seaweed.

Further, the isolation of the proteinaceous compound suggested the consideration of aqueous extract. Based on anti-quorum activity and phytochemical testing, *C. carandas* was selected for further studies. These activities of plants are due to their phytochemicals. The ability of the phytochemicals to affect the quorum-sensing behavior that plays a vital role in biofilms formation can be one of the explanations for the antibiofilm activity of the plant extracts. These phytochemicals affect the bacterial surface hydrophobicity and modify the cell permeability. Apart from this, phytochemicals also affect cellular motility, intercellular communication, and amino acid biosynthesis. Some phytochemicals like terpenoids, phenols, and alkaloids are able to affect the microbial enzymes and peptides of the cell membrane, which in turn causes cell disruption and eventually leads to cell death (Mostafa et al., 2018). Phytochemical screening of *C. carandas* shows the presence of flavonoids, phytosterols, phenols, terpenoids, protein, and saponins in plant extracts. Sánchez et al. (2016), during the phytochemical testing of three plants, *viz.*, *Prosopis laevigata*, *Opuntia ficus-indica*, and *Gutierrezia microcephala*, reported that these tested plants mainly contain steroids, flavonoids, coumarins, and tannins prima. Similarly, Monte et al. (2014) concluded in their work that 7-hydroxycoumarin (7-HC) and indole-3-carbinol (I3C) are the phytochemicals from plants that were found effective against *E. coli* and *S. aureus*.

Biofilm inhibition can be visualized by various techniques. In the performed study, bright-field microscopy was used to observe the inhibitory effect of the plant protein on the developed biofilm on a coverslip in 16-well plates. Sandberg et al. (2008) studied the inhibition of *S. aureus* using 96-well microtiter plates. Similarly, Merritt et al. (2011) used different solvents for different microbial biofilm solubilization with 0.1% CV dye in 96-well microtiter plates. Al-Sohaibani and Murugan (2012) studied the inhibition of biofilm development by *Salvadora persica* isolates against streptococcus biofilm. Wolf et al. (2002) used light microscopy to study the *E. coli*, *Staphylococcus epidermidis*, *P. aeruginosa*, and *Candida albicans* microbes biofilm morphology attached to the surface and the number of microbes that adhered to the surface. Lizana et al. (2013) used a microscope to analyze the biofilm structure before and after treatment with different plant extracts.

The EPS are comprised of protein substances, polysaccharides, glycolipids, and bacterial eDNA, which help the biofilm to become densely attached to the surfaces of catheters or wound beds. EPS degradation is one of the promising ways to destabilize microbial biofilms. Santhakumari et al. (2016) observed 66% and 68% inhibition of the biofilm formation and EPS production of the *Vibrio harveyi* and *Vibrio vulnificus* biofilm by the extract of unicellular algae *Synechococcus* sp. *C. violaceum* CV026 is used specifically for the study of the QQ nature of the compounds (Chang et al., 2019). Homoserine lactones are the major signaling molecules responsible for the biofilm formation in the *C. violaceum* CV026 and the gram-negative bacteria (Guzman et al., 2020). From this, we can speculate that the isolated plant protein might show effective binding affinity with the signaling molecule that results in the inhibition effect of the biofilm.

## Conclusion

An exponential increase in medical cases associated with biofilm infections causing a pandemic worldwide needs new antimicrobial compounds having low cross-resistance rates and potentially safe from cytotoxicity. The protein fraction isolated from the aqueous extract of *C. carandas* was found to possess promising anti-quorum-sensing activity. Further, this plant protein shows no cytotoxic effect on cells. The plant protein aqueous extract was found to have good activity at pH 7, incubation temperature at 4°C. It is the first report to demonstrate the anti-quorum-sensing activity of *C. carandas* plant aqueous extract, which is likely due to the presence of protein moiety present in it. The results of this plant-originated QQ-guided anti-biofilm study can serve as a benchmark for preparing new anti-infective leads for drugs in different formulations like ointments and mouthwashes to treat various chronic wound biofilms, biofilm-associated infections, and other oral diseases.

## Data Availability Statement

The original contributions presented in the study are included in the article/**Supplementary Material**. Further inquiries can be directed to the corresponding authors.

## Author Contributions

Conceived and designed the study and experiments: MS, VS, HH, MA, ML, YH, MM, MP, AM, BM, and SH. Performed the experiments: MS, VS, HH, ML, YH, and MM. Analyzed the data: MS, VS, HH, MA, ML, YH, and MM. Contributed reagents/materials/analysis tools: VS, MA, ML, MM, MP, AM, BM, and SH. Wrote the paper: MS, VS, HH, and SH. All authors reviewed the manuscript.

## Funding

MA would like to express his gratitude to AlMaarefa University, Riyadh, Saudi Arabia, for providing funding (TUMA-2021-53) to do this research.

## Conflict of Interest

The authors declare that the research was conducted in the absence of any commercial or financial relationships that could be construed as a potential conflict of interest.

## Publisher’s Note

All claims expressed in this article are solely those of the authors and do not necessarily represent those of their affiliated organizations, or those of the publisher, the editors and the reviewers. Any product that may be evaluated in this article, or claim that may be made by its manufacturer, is not guaranteed or endorsed by the publisher.
