# Supplementary material for: Quorum Quenching-Guided Inhibition of Mixed Bacterial Biofilms and Virulence Properties by Protein Derived From Leaves of Carissa carandas
Source: Front Cell Infect Microbiol. 2022 Jul 14;12:836819. doi: 10.3389/fcimb.2022.836819 (PMC9329584; doi:10.3389/fcimb.2022.836819)
Supplement: Supplementary file 1 [file DataSheet_1.pdf]

SUPPLEMENTARY INFORMATION

Quorum quenching guided inhibition of mixed bacterial biofilms and virulence properties by protein derived from leaves of *Carissa carandas*

Table SI1. Anti-quorum sensing activity of methanolic and aqueous plant extracts against bioindicator strain *Chromobacterium violaceum*

S. No.	Botanical name	Common name	Plant part	ZOI Methanolic extract (mm)	ZOI Aqueous extract (mm)
1	*Brassica nigra*	Mustard seeds	Seed	14	13
2	*Ricinus communis*	Castor	Seed	15	35
3	*Aegle marmelos*	Indian bael	Leaf	10	20
4	*Ficus religiosa*	Peepal	Leaf	10	13
5	*Murraya koenigii*	Curry patta	Leaf	12	20
6	*Psidium guajava*	Guava	Leaf	15	17
7	*Piper betle*	Paan	Leaf	14	20
8	*Ocimum tenuiflorum*	Holy basil	Leaf	-	20
9	*Tinospora cordifolia*	Giloy	Stem	17	22
10	*Lawsonia inermis*	Henna	Leaf	12	20
11	*Phyllanthus emblica*	Amla	Leaf	24	18
12	*Kigelia africana*	Barhal	Leaf	17	9
13	*Syzygium cumini*	Jamun	Leaf	19	15
14	*Carissa carandas*	Karonda	Leaf	10	20
15	*Butea monosperma*	Palash	Leaf	16	-
16	*Elettaria cardamomum*	Cardamom	Seed	15	13
17	*Mangifera indica*	Mango	Leaf	-	20
18	*Trachyspermum ammi*	Ajwain	Seed	20	18
19	*Allium sativum*	Garlic	Bulb	35	40
20	*Syzygium aromaticum*	Clove	Dry flower bud	40	20
21	*Curcuma longa*	Turmeric	Root	15	30
22	*Musa paradisiaca*	Banana	Leaf	15	-
23	*Neolamarckia cadamba*	Kadamb	Leaf	15	-
24	*Allium cepa*	Onion	Leaf	15	30
25	*Cinnamomum tamala*	Tejpatta	Leaf	17	16
26	*Cuminum cyminum*	Jeera	Seed	20	15
27	*Vigna angularis*	Adzuki	Seed	15	10
28	*Cinnamomum verum*	Dalchini	Bark	20	15

Table SI2. List of phytochemicals present in methanolic plant extracts

S. No	Phytochemical Plants	Car	Flv	Gly	Phe	Cou	Tan	Ter	Qui	Prt	Ps
1	*Brassica nigra*	+	+	+	+	+	+	+	-	+	+
2	*Ricinus communis*	-<br>+	-<br>+	+<br>+	+<br>+	+<br>+	-<br>+	+<br>-	-<br>-	-<br>+	+<br>-
3	*Aegle marmelos*	+	+	+	+	+	+	+	+	+	+
4	*Ficus religiosa*	+	+	-	+	+	+	-	-	+	-
5	*Murraya koenigii*	-	+	-	+	+	+	-	-	+	+
6	*Psidium guajava*	+	+	+	+	+	+	+	+	-	+
7	*Piper betle*	+	+	+	+	+	+	++	-	+	-
8	*Ocimum tenuiflorum*	+	+	+	-	+	+	-	-	+	+
9	*Tinospora cordifolia*	+	+	-	-	+	-	+	-	+	+
10	*Lawsonia inermis*	+	+	+	+	+	+	+	+	-	+
11	*Phyllanthus emblica*	-	+	+	+	+	+	-	+	+	+
12	*Kigelia africana*	+	+	+	+	-	+	+	+	-	+
13	*Syzygium cumini*	-	+	+	+	+	+	+	+	+	+
14	*Carissa carandas*	+	+	+	+	-	+	+	-	+	+
15	*Butea monosperma*	+	+	-	+	-	+	+	+	-	+
16	*Elettaria cardamomum*	+	+	+	-	-	-	+	-	+	-
17	*Mangifera indica*	-	+	+	+	+	+	+	+	-	-
18	*Trachyspermum ammi*	+	+	-	+	+	+	-	-	+	+
19	*Allium sativum*	-	+	+	+	+	+	+	+	-	+
20	*Syzygium aromaticum*	+	+	+	+	+	+	+	+	-	+
21	*Curcuma longa*	+	+	+	+	+	+	+	+	-	+
22	*Musa paradisiaca*	+	+	+	+	+	+	-	+	-	-
23	*Neolamarckia cadamba*	+	+	+	+	+	+	+	+	-	+
24	*Allium cepa*	+	+	+	+	-	+	-	-	+	+
25	*Cinnamomum tamala*	+	+	+	+	+	+	+	-	+	+
26	*Cuminum cyminum*	-	+	-	+	+	-	+	-	-	+
27	*Vigna angularis*	-	-	-	-	-	-	+	+	-	+
28	*Cinnamomum verum*	+	-	+	+	-	+	-	-	+	+

Car: Carbohydrate, Flv: Flavonoid, Gly: Glycoside, Phe: Phenol, Cou: Coumarin, Tan: Tannin, Ter: Terpenoid, Qui: Quinone, Prt: Protein, Ps: Phytosterol
